# Pathological Examination of the Placenta in COVID-19–Positive Mothers: A Short Communication

**DOI:** 10.30699/IJP.2023.551018.2860

**Published:** 2023-06-20

**Authors:** Sedigheh Hantoushzadeh, Roya Saeednejad, Mamak Shariat, Raheleh Moradi

**Affiliations:** 1 *Maternal, Fetal and Neonatal Research Center, Institute of Family Health, Tehran University of Medical Sciences, Tehran, Iran*; 2 *Breastfeeding Research Center, Institute of Family Health, Tehran University of Medical Sciences, Tehran, Iran*

**Keywords:** COVID-19, Pathology, Placenta, Pregnancy

## Abstract

**Background & Objective:**

It was declared that COVID-19 might be more severe in symptomatic pregnant patients. This study was conducted to examine the pathological indices of the placenta in pregnant women who were diagnosed with COVID-19.

**Methods:**

A total of 20 COVID-19–positive mothers were enrolled in this study. Detailed placental pathology findings were compared between subjects based on the history of abortion or occurrence of preterm delivery, hypertension, and diabetes.

**Results and Conclusion:**

Intervillositis was the most frequent abnormality of the placenta. There was also a significant association between abortion history and maternal vascular malperfusion (MVM; *P*=0.02). The placental abnormalities were found to be increased in women with COVID-19, regardless of maternal comorbidities. Further studies are needed to compare the placental pathology between COVID-19–positive women and healthy women.

## Introduction

COVID-19, caused by SARS-CoV-2, has spread worldwide since March 2020. Although a recent meta-analysis with data from over 435 infected pregnant women has shown that the severity of COVID-19 in pregnant women is not significantly different from nonpregnant women (1), in some studies, it has been declared that symptomatic pregnant patients are at higher risk of more severe infection compared to nonpregnant women of childbearing age (2,3).

Antepartum infection with fetal risks is one of the indications for the placental pathological examination (4). In some studies that have examined the placental pathology in women with COVID-19, fibrin deposition, vascular malperfusion, or thrombosis were common findings (5-7). In many studies, vertical transmission of SARS-CoV-2 from mother to fetus has been assessed by examination of the placental specimens, but positive cytogenetic results have been proven only in some of them (8-10). This study presents the results of a pathological examination of the placenta in COVID-19–positive mothers.

## Material and Methods

The study protocol was approved by the Ethics Committee of Tehran University of Medical Sciences (code: IR.TUMS.VCR.REC.1399.164). A total of 20 mothers with COVID-19 who were delivered at Vali-e-Asr Hospital of Tehran were enrolled in this study from March to September 2020. COVID-19 was definitely diagnosed based on the polymerase chain reaction (PCR) technique. Informed consent was obtained from all participants prior to their participation in the study. Maternal, perinatal, and placental features of the participants were prospectively collected (Tables 1, 2). 

Immediately after birth, the placental and membranous samples fixed in 10% buffered formalin were sent to the pathology laboratory. Then, the sections were stained with hematoxylin and eosin (H&E) staining. All placentas were examined for gross and histologic findings according to the Amsterdam Consensus Statement guidelines (11). 

SPSS version 23 (SPSS Inc., Chicago, IL., USA) was used to analyze the data, and P-values less than 0.05 were considered statistically significant. Detailed placental pathology findings were compared between subjects based on the history of abortion or occurrence of preterm delivery, hypertension, and diabetes using Fisher’s exact test. 

## Results and Discussion

The results of the pathological examination of the placenta showed that 8 out of the 20 subjects, were symptomatic. All women were in the third trimester of pregnancy, except one who was a candidate for termination due to fetal death at 19 weeks. The mean age of the subjects was 30.70±4.53 years, with a mean gestational age of 252.20±34.38 days. 

Of the 20 pregnancies, 19 neonates were born whose mean birth weight was 2857.37±730.77 g. The data on pregnancies are presented in Table 1. The mean placental weight and length of the umbilical cord were 468.47±158.87 g and 29.33±8.31 cm, respectively. In the third trimester, the maximum and minimum placental weights were 796 and 300 g, respectively. The longest cord was 47 cm, while the shortest one was only 16 cm. Table 2 shows a summary of the pathologic diagnoses. 

**Table 1:** The pregnancy profile and perinatal outcomes

Maternal age (year)	<18 or >35	4 (20%)	Maternal diabetes	Yes	2 (10%)
	18-35	16 (80%)		No	18 (90%)
Gestational age (week)	<37	6 (30%)	**Outcome of pregnancy**	Live birth	19 (95%)
	≥37	14 (70%)		stillbirth	1 (5%)
Delivery type	NVD^*^	7 (35%)	**Birth weight**	<2500	5 (26.3%)
	CS^**^	13 (65%)		≥2500	14 (73.7%)
Abortion history	Yes	5 (25%)	**PCR** ^***^ ** results in neonates**	Positive	3 (15.8%)
	No	15 (75%)		Negative	16 (84.2%)
Preterm delivery	Yes	1 (5%)	**NICU** ^****^ ** admission**	Yes	13 (68.4%)
	No	19 (95%)		No	6 (31.6%)
Maternal hypertension	Yes	0	**Neonatal survival**	Yes	18 (94.7%)
	No	0		No	1 (5.3%)

*Normal Vaginal Delivery

**Cesarean Section

***Polymerase Chain Reaction

****Neonatal Intensive Care Unit

**Table 2:** The pathological findings

Case	Histology of FVM^*^	Other Findings
1	None	MVM^**^, decidual arteriopathy
2	None	MVM, velamentous insertion
3	None	MVM, retroplacental hematomas
4	None	Intervillositis, deciduitis
5	None	-
6	None	-
7	Intramural fibrin deposition	Intervillositis, retroplacental hematomas, deciduitis, chorioamnionitis
8	Thrombosis	-
9	None	Retroplacental hematomas, chorangiosis
10	None	-
11	None	Intervillositis, deciduitis
12	Thrombosis	Retroplacental hematomas
13	Thrombosis	Intervillositis
14	Thrombosis	-
15	None	MVM, intervillositis, deciduitis
16	Thrombosis	-
17	None	-
18	Thrombosis	Intervillositis
19	None	Intervillositis
20	None	Chorioamnionitis

*Fetal Vascular Malperfusion

**Maternal Vascular Malperfusion

Intervillositis, thrombosis, and maternal vascular malperfusion (MVM) were the most common findings, but none of them have been seen in normal pregnancies (12). In a pathological assessment of the placenta of the 20 COVID-19–positive subjects, fetal vascular malperfusion (FVM) or fetal vascular thrombosis was shown in 10 subjects (6). Shanes *et al*. examined histopathologic findings in the placentas of women with COVID-19 compared with historical controls and women with the placental evaluation for a history of melanoma (7). Compared to controls, third-trimester placentas were significantly more likely to show at least 1 feature of MVM (particularly abnormal or injured maternal vessels) and intervillous thrombosis. Further studies are needed to determine whether the thrombosis and vascular malperfusion in some of the cases described in these studies are related to hypercoagulability associated with COVID-19 and whether intervillositis of unknown etiology is related to an antiviral immune response.

While there was no significant association between the placental abnormalities and obstetric complications (including preterm delivery, maternal hypertension, or diabetes), it was seen a significant difference in MVM between the 2 groups based on the history of miscarriage (*P*=0.02). Therefore, vascular malperfusion increased in women with at least 1 history of abortion. Malperfusion of maternal vessels refers to the microscopic findings of the placental pathology, often leading to hypoplasia-included decidual arteriopathy, infarction, increased perivillous fibrin, accelerated villous maturation, and distal villous hypoplasia that may be associated with a fetal loss (13).

In this study, 3 newborns were definitely diagnosed with COVID-19, though none of the placental cytogenetic results were positive by PCR. In the study by Yang and Liu, the vertical transmission of SARS-CoV-2 was evaluated by reviewing a total of 22 studies comprising 83 neonates born to mothers with COVID-19. Among these neonates, 3 were confirmed with SARS-CoV-2 infection 16, 36, and 72 h after birth. In all 22 studies, the PCR tests of the placenta, amniotic fluid, or cord blood were negative, and there was a lack of virological evidence for intrauterine vertical transmission (14). In the review of Deniz and Tezer, 50 studies (including 17 newborns testing positive for SARS-CoV-2) were reported (8). Eight placental tissues, 3 breast milk samples, and 1 amniotic fluid test were positive by PCR; this result supports vertical transmission of SARS-CoV-2. In a recent report, Zaigham *et al.* examined virus isolates from the mother and neonate, as well as from the placental tissue, for whole genome sequencing. Transplacental transmission, followed by the similarity of the viral genome of samples, was confirmed (10). In our study, the negative PCR results of placental specimens cannot rule out the possible vertical transmission of SARS-CoV-2, and further studies with additional cases are needed in this regard. In addition to the incidence of chorioamnionitis in one COVID-19–positive neonate can discuss the odds of microbial intrauterine transmission and the operation of the genome mapping techniques may help to prove vertical transmission of the virus.

## Conclusion

The placental abnormalities were found to be increased in women with COVID-19, regardless of maternal comorbidities. Although the placental injury was retrospectively compared between a cohort of COVID-19 and negative controls (15), more comparative studies are needed to analyze the difference in the placental pathology based on the diagnosis of COVID-19 or being symptomatic.

## Conflict of Interest

The authors declare no conflict of interest.
